# Ghrelin is a persistent biomarker for chronic stress exposure in adolescent rats and humans

**DOI:** 10.1038/s41398-018-0135-5

**Published:** 2018-04-11

**Authors:** Muhammad I. ul Akbar Yousufzai, Elia S. Harmatz, Mohsin Shah, Muhammad O. Malik, Ki A. Goosens

**Affiliations:** 1 Department of Physiology, Institute of Basic Medical Sciences, Khyber Medical University, Peshawar, Pakistan; 2 McGovern Institute for Brain Research & Department of Brain and Cognitive Sciences, 77 Massachusetts Ave, Cambridge, MA 02139 USA; 3 Department of Neurology, Massachusetts General Hospital, 114 16th Street, Charlestown, MA 02129 USA

## Abstract

Prolonged stressor exposure in adolescence enhances the risk of developing stress-sensitive mental illnesses, including posttraumatic stress disorder (PTSD), for many years following exposure cessation, but the biological underpinnings of this long-term vulnerability are unknown. We show that severe stressor exposure increased circulating levels of the hormone acyl-ghrelin in adolescent rats for at least 130 days and in adolescent humans for at least 4.5 years. Using a rodent model of longitudinal PTSD vulnerability in which rodents with a history of stressor exposure during adolescence display enhanced fear in response to fear conditioning administered weeks after stressor exposure ends, we show that systemic delivery of a ghrelin receptor antagonist for 4 weeks surrounding stressor exposure (2 weeks during and 2 weeks following) prevented stress-enhanced fear memory. These data suggest that protracted exposure to elevated acyl-ghrelin levels mediates a persistent vulnerability to stress-enhanced fear after stressor exposure ends.

## Introduction

A history of stressor exposure is a risk factor for the later development of disease^1^. This includes post-traumatic stress disorder (PTSD) following trauma exposure in rodent models of PTSD^2,3^ and humans^4–6^. Whether the stressor exposure occurs in adolescence or adulthood, the elevated risk of PTSD persists long beyond the presence of the stressor. In rodents, stress-enhanced fear learning is a model of PTSD vulnerability in which intense stressor exposure leads to enhanced fear memory strength in a Pavlovian fear conditioning task^2^ even when fear conditioning occurs weeks following stressor cessation^7^. In this rodent model, persistent changes in fear circuitry have been observed for weeks after stressor exposure^2,8,9^. In humans, stress-enhanced risk of PTSD has been observed decades following stressor exposure^4–6,10,11^.

The mechanisms underlying such a persistent vulnerability are unknown. We recently reported that chronic stress elevates peripheral circulating acyl-ghrelin^2,3^. We found that acyl-ghrelin was elevated only by stressor exposures that were sufficient to produce stress-enhanced fear learning^2^. This elevated signaling via acyl-ghrelin drives stress-enhanced fear learning in rats when trauma occurs in the days following stressor cessation: systemic administration of a ghrelin receptor antagonist during chronic stress exposure prevented stress-enhanced fear in the days immediately following stressor cessation^2^. However, other studies reported that acyl-ghrelin remains elevated for at least 30 days following stressor cessation in rodents^12,13^. This raises the possibility that stress-induced elevations of ghrelin may contribute to an increased risk of stress-enhanced fear learning when trauma occurs long after stressor exposure ends.

While acyl-ghrelin levels have been shown to remain elevated for at least 30 days post-stressor exposure in adult rodents, no studies have examined acyl-ghrelin levels following adolescent stress at any time point. In addition, no studies have examined acyl-ghrelin levels in humans following severe stressor exposure. Here, we sought to examine acyl-ghrelin levels at remote time points following severe stressor exposure in adolescent rats and humans. We report that chronic stressor exposure elevated acyl-ghrelin for at least 130 days in rats and 4.5 years in adolescent humans. We then sought to provide proof-of-principle evidence linking elevated ghrelin to stress-enhanced fear at a time point remote to stressor exposure in an adolescent rodent model of stress-enhanced fear learning. We show that elevated acyl-ghrelin signaling after chronic stressor exposure was sufficient to produce stress-enhanced fear learning, a rodent model of PTSD vulnerability, in adolescent rats. Furthermore, we show that prolonged reduction of acyl-ghrelin signaling throughout stressor exposure and until fear conditioning completely prevented stress-enhanced fear. Our data suggest that chronic stress exposure increases the risk of PTSD by elevating acyl-ghrelin for an extensive period following the initial occurrence of the primary stressor.

## Materials, subjects and methods

Additional details for all procedures are included in the Supplementary Materials and Methods.

### Rodent subjects

All rodent experiments used adolescent, male Long Evans rats (225–250 g, ~6 weeks of age at the start of the experiment; Charles River, Raleigh, NC), individually housed (20–22.2 °C, 12-h light/dark cycle, 0700 h lights on). All procedures involving rodents were approved by the Institutional Animal Care and Use Committee of the Massachusetts Institute of Technology and the Animal Care and Use Review Office of the US Army Medical Research and Materiel Command, and complied with federal, state, and local laws concerning the use of vertebrate animals in research.

### Human subjects

Participants were recruited from the local population through “Jirga” (local councils of elders) in the northwest part of Khyber Pukhtunkhwa province, a geographic locale affected by terrorist activities for more than a decade. Children in the Traumatized group were defined as those who lost a loved one or who were themselves injured in terror attacks. Children in the Control group must never have lost a loved one or been injured in a terror attack. After exclusions (see Study Inclusion and Exclusion Criteria below), 88 children [Traumatized: *n* = 49 (46 male and 3 female) from 35 families; Control: *n* = 39 (39 male and 0 female) from 33 families] were included in the study. The study was approved by the Institutional Review Board of Khyber Medical University, Pakistan. After the nature of the study was explained in detail, informed consent was obtained from all participants and their caregivers.

### Drug preparation

For systemic drug delivery, rats were injected with 1 mL kg^−1^ (i.p.) of either vehicle or drug. D-Lys^3^-GHRP-6 (DLys; Tocris Biosciences; Minneapolis, MN) was diluted to 5.48 µg/mL in sterile saline. DLys is a blood–brain barrier-penetrant^14^ inhibitor of GHSR1a,^15^ which also exhibits activity at chemokine receptors^16,17^.

### Chronic immobilization stress

Some rats received immobilization stress from 11:00 a.m. to 3:00 p.m. in Decapicone plastic bags (Braintree Scientific; Braintree, MA), which were secured at the tail. Immobilized rats were secured in pairs to the floor of cages lacking bedding for 14 consecutive days.

Unstressed control rats were handled daily for 1 min.

### Rodent venous blood collection

All blood collection occurred between 1 and 3 PM, as previously described^18^.

### Human venous blood collection

Acyl-ghrelin was measured in serum samples by the method of Blatnik et al.^19^, with some modifications described in Supplementary Materials and Methods. All samples were collected at approximately 5:00 p.m.

### Human clinical evaluation

A structured questionnaire was used in a face-to-face interview with children and their caregivers. Questionnaires were administered in Urdu and were translated to English for inclusion in this manuscript (see Supplementary Materials, Questionnaire S1). Translation was performed by four people from Khyber Medical University fluent in both Urdu and English. This information was collected either prior to or after the collection of the venous blood sample. Importantly, because acute stressor exposure does not alter circulating acyl-ghrelin levels^3^, the precise timing of the questionnaire should not have altered the measurement of acyl-ghrelin.

### Hormone assays

Acylated ghrelin levels were measured with commercial ELISA kits. For rat plasma samples, undiluted, acidified samples were processed according to the manufacturer’s protocol (Millipore; Billerica, MA).

For human serum samples, acyl-ghrelin levels were determined with an ELISA kit specific to human acyl-ghrelin (Glory Science Co., Ltd.; Product No. 12572; Del Rio, TX) according to the manufacturer’s instructions.

No samples displayed signs of hemolysis or lipemia. For all ELISAs, the samples were processed and analyzed by people who were not involved in sample collection and blind to the status of each sample. Samples were run in duplicate and mean values were used in subsequent analyses. All reported values were dilution-corrected.

### Rodent fear conditioning

Some rats, as described in the main text, were subjected to auditory Pavlovian fear conditioning and fear recall testing. All rats were transported in their home cages from the vivarium to a holding room in which no behavioral testing was conducted. This transport occurred at least 1 h prior to the onset of any behavioral testing.

### Statistical analyses

Statistical analyses were carried out using SPSS 20. Summary statistics were calculated and data were expressed as means ± standard error. The normality of the data was tested using Kolmogorov–Smirinov and Shapiro–Wilk tests and histograms. Study inclusion and exclusion criteria are described in the Supplementary Materials and Methods. To meet normality requirements for statistical tests, the values for “Age at Study Initiation” were squared, and the values for acyl-ghrelin were logarithmically transformed (log_10_) before statistical testing. Independent sample *T*-tests were used to examine the differences between the Control and Traumatized groups. Comparison of categorical data between Control and Traumatized groups were analyzed using *χ*^2^ tests. Univariate and multivariate regression analyses were done to investigate the association of ghrelin with several variables. A *p*-value of less than 0.05 was considered significant.

## Results

### Chronic stressor exposure in adolescent rats produces a long-term increase in circulating acyl-ghrelin

Previous studies showed that chronic stress in adult rodents produced a robust increase in circulating acyl-ghrelin measured 1 day after the last stressor exposure; this increase persisted at least 4 weeks after the last stressor exposure^12,13^. Here, we sought to extend these findings to stressor exposure beginning in adolescence using time points even more remote than those previously assessed.

**Fig. 1:**
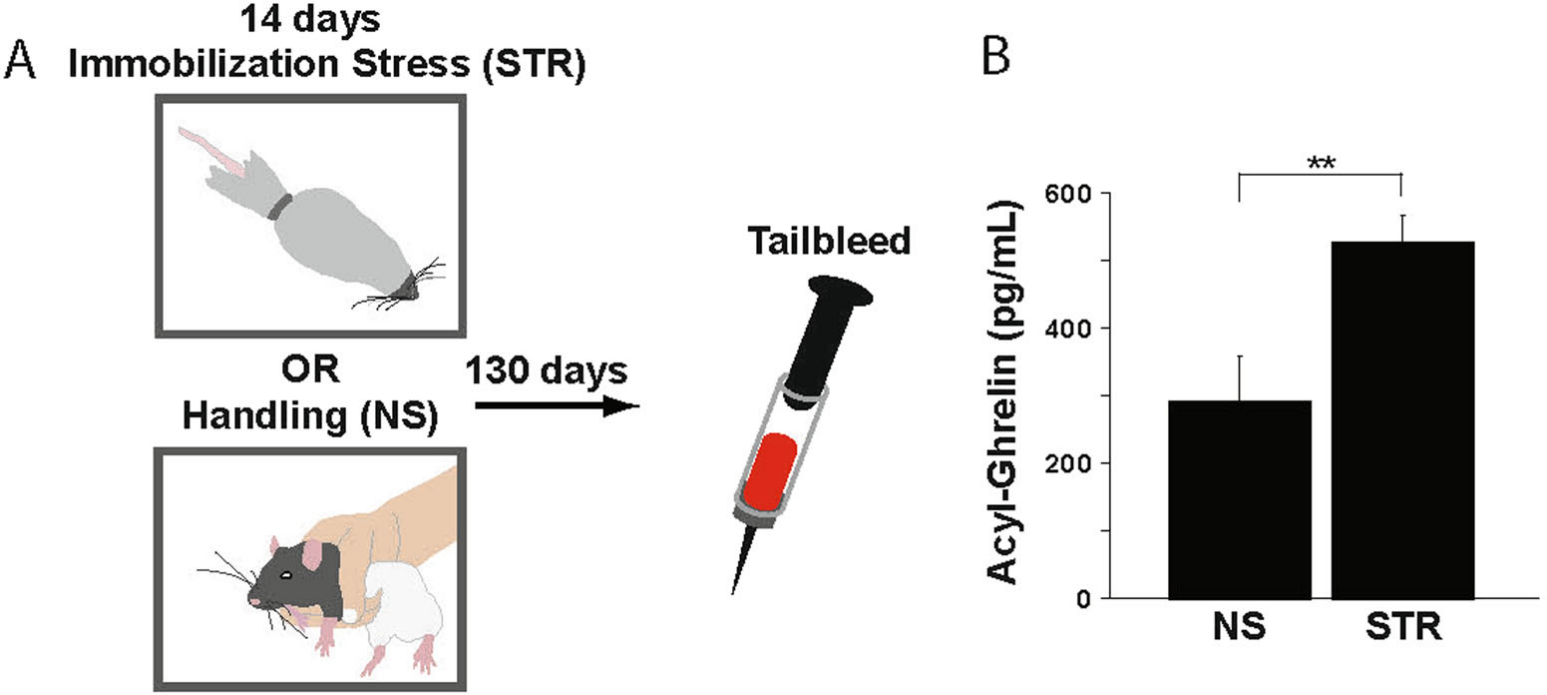
Chronic stress in adolescent rats elevates acyl-ghrelin for at least 130 days post-stressor cessation. **a** Experimental design. Rats received either handling (NS no stress, group; *n* = 10) or immobilization stress (4 h/day; Stress, or STR, group; *n* = 26) for 14 days. All rats remained in their home cages without further manipulation for 130 days. Venous blood samples were collected at this time point. **b** Acyl-ghrelin was measured in the blood samples. All values are ± SEM. *p < 0.05 in a post-hoc comparison

Adolescent rats were exposed to two weeks of immobilization stress or daily handling starting at approximately postnatal day 50, a period corresponding to rodent adolescence^20^. Body weights did not differ between the two groups at the start of the experiment (data not shown; main effect of group: *F* (1,34) = 0.32, *p* = 0.58). Twenty-four hours after the last stress or handling session, acyl-ghrelin was significantly elevated in the chronically stressed rats (Fig. S1; main effect of group: *F*(1,23) = 33.00, *p* < 0.0001), an effect similar to that reported for stress-exposed adult rats^2,3^. One hundred and thirty days following the final stress or handling session (Fig. 1a), an elapsed time corresponding roughly to 12 human years^21^, acyl-ghrelin remained significantly elevated in the rats in the STR group (Fig. 1b; main effect of group: *F*(1,34) = 9.63, *p* = 0.0038). Thus, chronic stressor exposure in adolescent rats produced a long-term elevation in circulating acyl-ghrelin that persisted long after the stressor was ceased.

### Trauma exposure produces a long-term increase in circulating acyl-ghrelin in adolescent humans

**Fig. 2:**
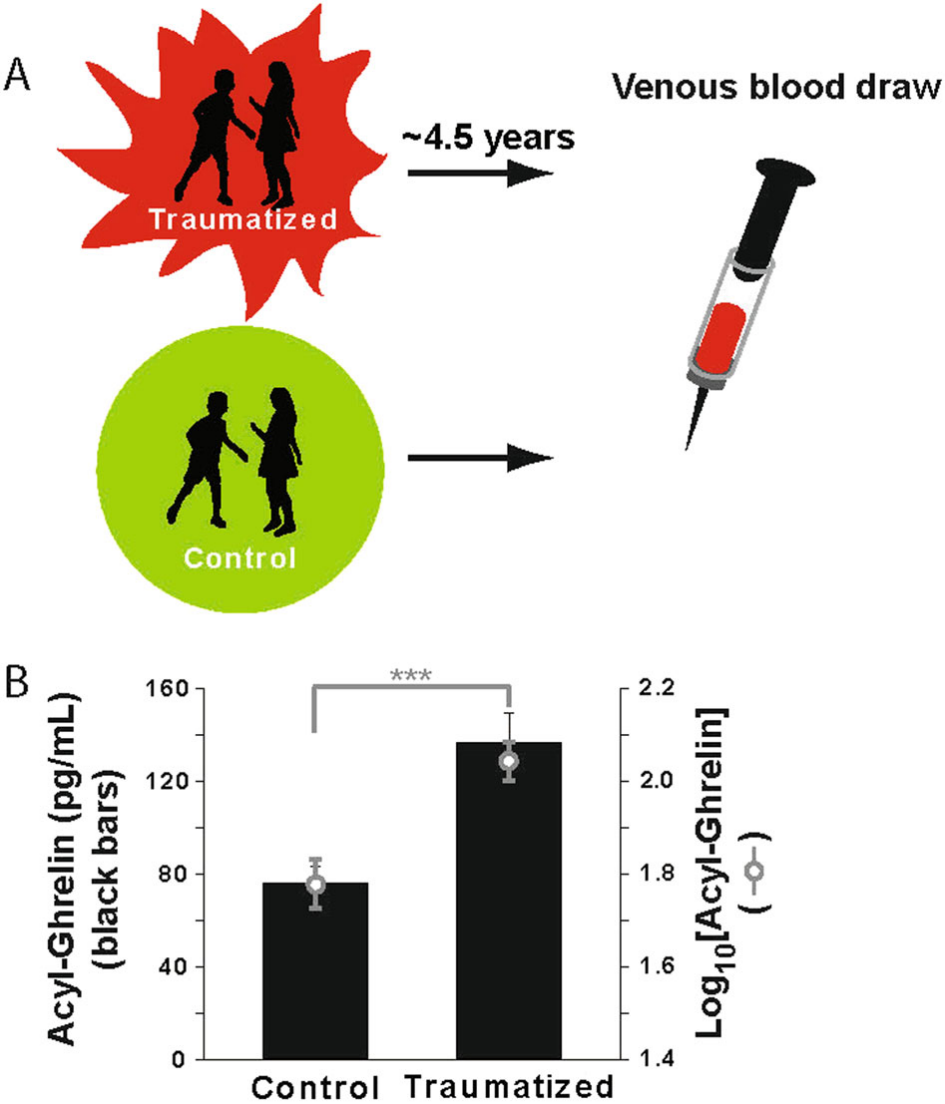
Terror-associated stressor exposure elevates acyl-ghrelin in adolescent human subjects. Venous blood samples were collected from adolescent subjects who were either injured or lost a loved one in a terror attack (Traumatized group; average of 4.49 ± 0.24 years between the attack and sample collection; *n* = 49) or adolescent subjects who had never been injured or lost a loved one in a terror attack (Control group; *n* = 39). Black bars indicated non-transformed mean values. Gray open circles indicate log-transformed mean values. All values are ± SEM. ***p < 0.001 in a post-hoc comparison of the log-transformed values

To determine whether acyl-ghrelin was elevated in adolescent humans following significant stressor exposure, we measured acyl-ghrelin in Pakistani children who were either injured or lost a loved one in a terror attack (Traumatized group) or children who had never been injured or lost a loved one in a terror attack (Control group) (Fig. 2a). Children were included in the study only if they were physically healthy and had no chronic health conditions (including previously diagnosed psychiatric disorders). For children in the Traumatized group, the average age at the time of trauma exposure was 9.8 ± 0.52 years. Children in the two groups were matched at the time of sample collection for age (average age: 14.31 ± 0.30 years), weight, and height (Table 1), as well as for gender and socioeconomic status (Table 2). Although children in the Traumatized group had a body mass index (BMI) that was significantly lower than that of children in the Control group (Table 2), the majority of children in both groups had a BMI within a healthy, normal range (Fig. S2A)^22^. Also, acyl-ghrelin levels did not vary with BMI classification either across or within groups (Fig. S2B; main effect of classification: *F*(2,82) = 0.082, *p* = 0.92, Classification × Group interaction: *F*(2,82) = 0.017, *p* = 0.98). The absence of a correlation between acyl-ghrelin and BMI may arise because our human subject population did not include any obese children. Obese children have high BMIs and low acyl-ghrelin levels^23^, the opposite to what is typically observed in children with a lean BMI.

**Table 1:** Anthropometric variables for all human study participants. All values are the arithmetic mean ± SEM

Characteristics	Total population (*n* = 88)	Control (*n* = 39)	Traumatized (*n* = 49)	*P*-value^a^
Age at study initiation (year)	14.31 ± 0.30 (*n* = 88)	14.33 ± 0.42 (*n* = 39)	14.29 ± 0.42 (*n* = 49)	0.98^b^
Height (m)	1.52 ± 0.02 (*n* = 88)	1.49 ± 0.03 (*n* = 39)	1.55 ± 0.02 (*n* = 49)	0.097
Weight (kg)	44.6 ± 1.5 (*n* = 88)	45.59 ± 2.30 (*n* = 39)	43.84 ± 2.03 (*n* = 49)	0.57
BMI	18.88 ± 0.37 (*n* = 88)	20.33 ± 0.53 (*n* = 39)	17.72 ± 0.47 (*n* = 49)	0.0004
Elapsed time since loss of closest loved one (year)^c^	2.62 ± 2.59 (*n* = 51)	5.5 ± 0.50 (*n* = 2)	4.49 ± 1.71 (*n* = 49)	0.42
Total loved ones who have died	1.94 ± 0.24	0.10 ± 0.07 (*n* = 39)	3.41 ± 0.29 (*n* = 49)	<0.0001

^a^ The *p*-value is for an independent sample *t*-test comparison between the two groups

^b^ Age was squared to meet the normality distribution requirements for statistical analysis

^c^ For children in the Traumatized group, the elapsed time reflects the years since the terror attack that resulted in the loss of a loved one or injury to the child. For children in the Control group, the elapsed time represents the years since the natural death of a loved one; the majority of subjects in this group had never lost a loved one, and were excluded from this analysis

**Table 2:** Comparison of variables between the Traumatized and Control groups

Variables	*n* (% within group)	Group *n* (% within group)		
		Control (*n* = 39)	Traumatized (*n* = 49)	*P*-value
Gender	Male	39 (100.0%)	46 (93.9%)	0.116
	Female	0 (0.0%)	3 (6.1%)	
Type of terror-associated trauma	School Massacre	N/A	2 (4.1%)	N/A
	Bomb blast	N/A	42 (85.7%)	
	Shooting	N/A	5 (10.2%)	
Closest loved one who has died	Father	0 (0.0%)	17 (34.7%)	<.001
	Mother	2 (5.1%)	2 (4.1%)	
	Sibling	0 (0.0%)	6 (12.2%)	
	Friend	0 (0.0%)	1 (2.0%)	
	Other Relative	0 (0.0%)	14 (28.6%)	
Injury to self in terror event	Yes	N/A	9 (18.4%)	<.001
	No	N/A	40 (81.6%)	
Socioeconomic status	Upper	1 (2.6%)	1(2.0%)	0.871
	Middle	10 (25.6%)	15 (30.6%)	
	Lower	28 (71.8%)	33 (67.3%)	
Medications taken at younger age	No	39 (100.0%)	49 (100.0%)	N/A
Medications taken in recent years	No	38 (97.4%)	48 (98.0%)	0.87
	Yes	1 (2.6%)	1(2.0%)	
Sleep pattern change/disturbance	No	32 (100.0%)	2 (4.1%)	<.001
	Yes	0 (0.0%)	47 (95.9%)	
Weight change	No	37 (94.9%)	3 (6.1%)	<.001
	Yes	2 (5.1%)	46 (93.9%)	
Periods of confusion	No	38 (97.4%)	1(2.0%)	<.001
	Yes	1 (2.6%)	48 (98.0%)	
Periods of non-stop crying	No	31 (96.9%)	3 (6.1%)	<.001
	Yes	1 (3.1%)	46 (93.9%)	
Concentration problems	No	31 (96.9%)	1(2.0%)	<.001
	Yes	1 (3.1%)	48 (98.0%)	
Not coping well with loss of loved one or injury	N/A	30 (93.8%)	0 (0%)	<.001
	No	2 (6.2%)	12 (25%)	
	Yes	0 (0%)	36 (75%)	
Suicidal thoughts	No	39 (100.0%)	26 (53.1%)	<.001
	Yes	0 (0.0%)	23 (46.9%)	
Thoughts of harming others	No	32 (100.0%)	12 (25.0%)	<.001
	Yes	0 (0.0%)	36 (75.0%)	
Decision power (disturbed)	No	30 (93.8%)	2 (4.2%)	<0.001
	Yes	2 (6.3%)	46 (95.8%)	
Behavior change	No	35 (89.7%)	1 (2.0%)	<0.001
	Yes	4 (10.3%)	48(98.0%)	
Rage	No	3692.3%)	0 (0.0%)	<0.001
	Yes	3 (7.7%)	49 (100.0%)	
Physical pain	No	31 (96.9%)	4 (8.5%)	<0.001
	Yes	1 (3.1%)	43 (91.5%)	
Crying and sighing	No	39 (100.0%)	2 (4.1%)	<0.001
	Yes	0 (0.0%)	47 (95.9%)	
Headache	No	37 (94.9%)	8 (16.7%)	<0.001
	Yes	2 (5.1%)	40 (83.3%)	
Appetite loss	No	38 (97.4%)	5 (10.2%)	<0.001
	Yes	1 (2.6%)	44 (89.8%)	
Difficulty sleeping	No	37 (94.9%)	4 (8.2%)	<0.001
	Yes	2 (5.1%)	45 (91.8%)	
Weakness	No	39(100.0%)	4(8.2%)	<0.001
	Yes	0 (0.0%)	45 (91.8%)	
Fatigue	No	36 (92.3%)	8 (16.3%)	<0.001
	Yes	3 (7.7%)	41 (83.7%)	
Feeling of heaviness	No	38 (97.4%)	13 (27.1%)	<0.001
	Yes	1 (2.6%)	35 (72.9%)	
Aches, pains	No	38 (97.4%)	10 (20.8%)	<0.001
	Yes	1 (2.6%)	38 (79.2%)	
Anxiety	No	37 (94.9%)	0 (0.0%)	<0.001
	Yes	2 (5.1%)	49 (100.0%)	
Frustration	No	37 (94.9%)	0 (0.0%)	<0.001
	Yes	2 (5.1%)	49 (100.0%)	
Guilt	No	36 (92.3%)	7 (14.3%)	<0.001
	Yes	3 (7.7%)	42 (85.7%)	
Isolation	No	36 (92.3%)	8 (16.3%)	<0.001
	Yes	3 (7.7%)	41(83.7%)	
Question reason for the loss or injury	No	36 (92.3%)	5 (10.4%)	<0.001
	Yes	3 (7.7%)	43 (89.6%)	
Life and death awareness	No	34 (87.2%)	0 (0.0%)	<0.001
	Yes	5 (12.8%)	49 (100.0%)	

Values indicate the number of participants (*n*) and percentage of participants (%) within each group. *P*-value is for *χ*^2^ tests between groups

*N/A* not applicable

Demonstrating the stressful impact of the terror attacks, children in the Traumatized group exhibited many differences in psychological function compared to children in the Control group, including differences in sleep, emotional regulation, and social isolation (Table 2). The children in the Traumatized group also exhibited roughly twice the level of circulating acyl-ghrelin as children in the Control group (Fig. 2b; *F*(1,86) = 16.62; *p* = 0.0001).

In a univariate regression analysis, acyl-ghrelin was significantly associated with group (*β* = 0.399, *p* = 0.001). In contrast, acyl-ghrelin levels were not related to age at the time of sample collection, BMI, elapsed time since the death of a loved one or injury to self, or socioeconomic status (Table S1). Similar results were seen with multivariate regression analysis (Table S1).

### Prolonged exposure to elevated ghrelin signaling underlies persistent vulnerability to stress-enhanced fear after stressor cessation

**Fig. 3:**
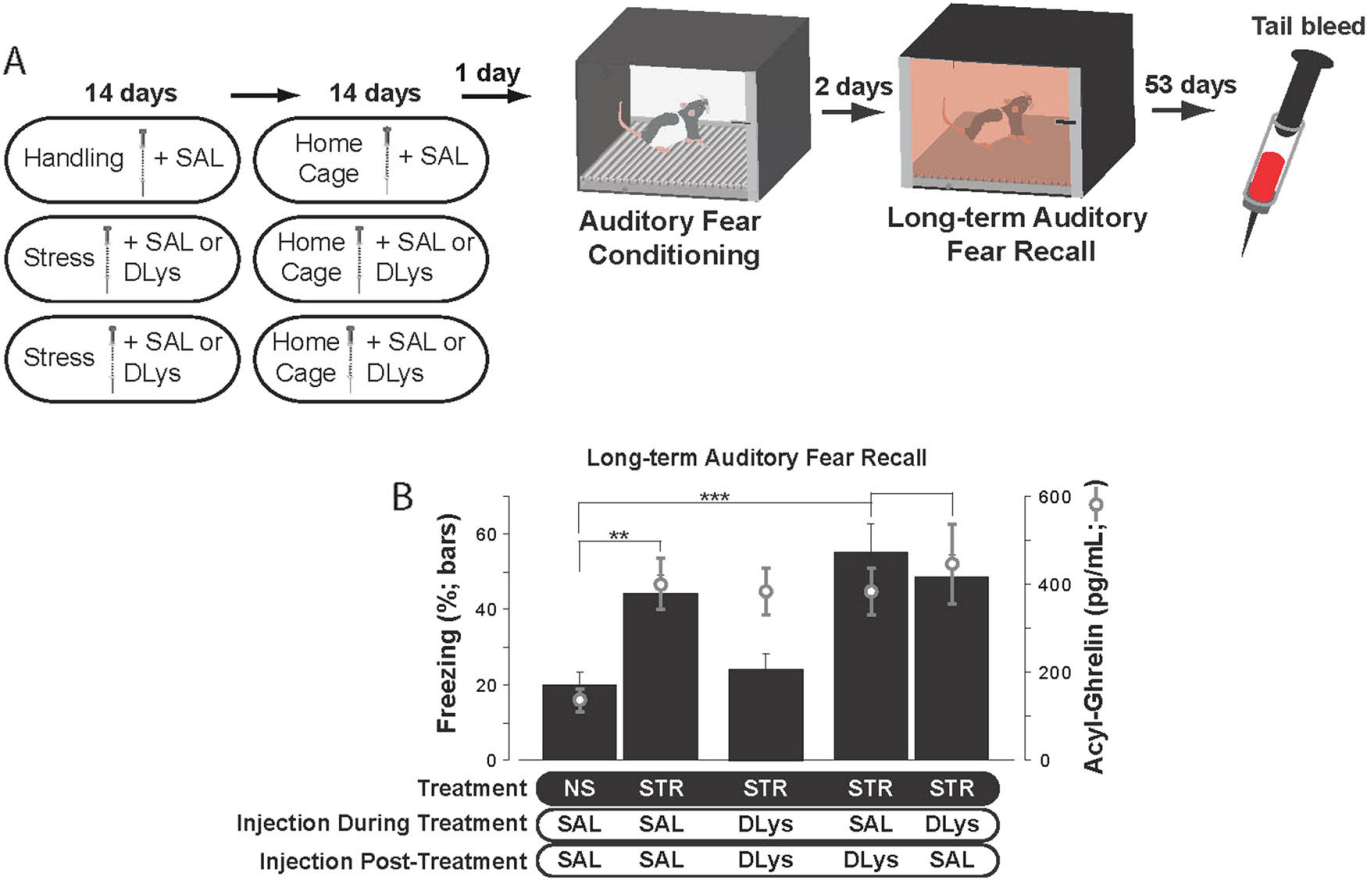
Elevated ghrelin signaling either during chronic stress or after the cessation of stress is sufficient to enhance subsequent fear learning. **a** Experimental design (*n* = 13 to 15/group). Rats received either handling (NS group) or immobilization stress (4 h/day; STR group) for 14 days. Rats received injections (i.p.) of either saline (SAL) or the ghrelin receptor antagonist D-Lys^3^-GHRP-6 (DLys). For 14 days after the last day of immobilization of handling, rats remained in their home cages and received injections of SAL or DLys. Twenty-four hours after the final injection, all rats were subjected to auditory Pavlovian fear conditioning. Two days later, long-term auditory fear recall was measured. Fifty-three days later (60 days after the last day of stressor exposure or handling), tail blood samples were collected. **b** Black bars indicate mean freezing levels during the tone presentations of the long-term auditory fear recall test. Gray open circles indicate acyl-ghrelin levels measured 60 days after the last stressor exposure or handling session. All values are ± SEM. **p < 0.01, ***p < 0.001 in indicated post-hoc comparisons

Our results show that chronic stress exposure produced an enduring elevation of acyl-ghrelin in the bloodstream of both rats and humans exposed to stressors as adolescents. This long-term dysregulation of acyl-ghrelin may therefore contribute to a stress-related vulnerability to PTSD that persists long beyond the initial stressor exposure^2–6^. To test this hypothesis, we used a rodent model of vulnerability to PTSD in which adolescent rats were either handled (No Stress, or NS, groups) or exposed to chronic immobilization stress (Stress, or STR, groups) (Fig. 3a)^2,3,24^. When auditory Pavlovian fear conditioning was administered 2 weeks after the cessation of stress or handling (Fig. S3), rats in the STR group displayed significantly enhanced long-term fear recall compared to rats in the NS group (Fig. 3b; STR–SAL–SAL vs. NS–SAL–SAL: *F*(1,25) = 13.00, *p* = 0.0014). Thus, prolonged stressor exposure starting in adolescence produced a vulnerability to enhanced memory for traumatic experiences, despite a temporal gap between the cessation of stressor application and subsequent trauma.

A ghrelin receptor antagonist delivered during 14 days of immobilization stress has previously been shown to be sufficient to prevent stress-enhancement of fear when Pavlovian fear conditioning was administered 1 day after the last day of immobilization stress^2^. Here, we found that if the ghrelin receptor antagonist D-Lys^3^-GHRP-6 (DLys) was administered during the two weeks of immobilization stress, but Pavlovian fear conditioning was administered at a remote time point (two weeks after the final immobilization exposure), stress-enhanced fear was no longer blocked: rats in the STR–DLys–SAL group exhibited significantly higher auditory fear recall compared to rats in the NS–SAL–SAL group (Fig. 3b; *F*(1, 25) = 14.16, *p* = 0.0009). Because systemic injection of DLys during chronic immobilization stress did not blunt the ability of chronic stress to elevate ghrelin (Fig. 3b; *F*(1,24) = 12.5, *p* = 0.0017), this finding suggests that the continued elevation of acyl-ghrelin beyond stressor cessation causes vulnerability to stress-enhanced fear to re-emerge when a ghrelin receptor antagonist is no longer administered.

To determine whether stress-enhanced fear at the remote 2-week time point could be blocked if ghrelin receptor activity was reduced throughout the entire period spanning stressor exposure through fear conditioning, the DLys was delivered daily during stressor exposure and in the two weeks after stressor cessation. In this case, stress-enhanced fear memory was completely blocked: rats in the STR-DLys-DLys group froze at levels similar to rats in the saline (SAL)-injected NS–SAL–SAL control group (Fig. 3b; *F*(1, 27) = 0.53, *p* = 0.47). Thus, despite having elevated levels of endogenous acyl-ghrelin (Fig. 3b; *F*((1, 27) = 16.2; *p* = 0.0004), reducing the actions of this ligand at its receptor throughout stressor exposure and until subsequent trauma exposure prevented stress-enhanced fear.

In contrast, a ghrelin receptor antagonist administered only in the 14-day period spanning stressor cessation through trauma exposure was not sufficient to prevent stress-enhanced fear: rats in the STR–SAL–DLys group exhibited significantly higher auditory fear recall compared to rats in the NS–SAL–SAL group (Fig. 3b; F(1,25) = 17.21, *p* = 0.0003). Thus, the stress-related enhancement in ghrelin that occurs during stressor exposure^2,3^ triggers a cascade of events that cannot be readily reversed by blocking ghrelinergic signaling after stressor exposure.

## Discussion

Prolonged exposure to stressors renders individuals vulnerable to PTSD when trauma is encountered later in life. This risk factor is particularly well-studied in adolescent humans, where childhood stressor exposure leads to enhanced PTSD prevalence when trauma is encountered in childhood^25,26^ or adulthood^27–29^. Childhood stressor exposure has also been associated with stronger symptoms of PTSD^28,30,31^. The existence of a temporal gap between stressor exposure and subsequent additional trauma suggests that there is a window of opportunity to reverse or modify the impact of stress on PTSD vulnerability, but understanding the mechanisms by which stress causes enhanced vulnerability to PTSD is essential for achieving this clinical goal.

Here, we focused on the role of acyl-ghrelin in this vulnerability. Prolonged stress enhances circulating acyl-ghrelin^32,33^ and this increase persists beyond the cessation of stressor exposure^12,13^. We extend these findings by showing that chronic stress starting in adolescence produces an elevation in acyl-ghrelin that lasts for months in rats (Fig. 1). Also, we provide the first evidence that acyl-ghrelin is similarly elevated by prolonged stressor exposure in human subjects (Fig. 2). Multiple biomarkers are associated with long-term physical and mental health outcomes in human subjects^34^. However, there is no single biomarker that is robustly altered over the long-term after stressor exposure ceases across human subjects. Our results suggest that acyl-ghrelin, when combined with other biomarkers, may enhance the ability to detect and measure chronic stressor exposure in human subjects.

In addition to PTSD vulnerability, chronic stress also enhances risk for other mental health disorders which can co-occur with PTSD. Of these, chronic stress and ghrelin have both been linked to major depressive disorder and alcohol use disorder (AUD). Chronic stress produces a prolonged elevation of ghrelin and a depressive phenotype in mice^12^. Because knockout of the ghrelin receptor deepens stress-induced depression^12^, this suggests that stress-induced increases in ghrelin may lead to a loss of ghrelin sensitivity in depression-related neural circuits, similar to what is reported for fear circuitry^3^. While some studies suggest that ghrelin levels are elevated in patients with AUD^35^, it is clear that administration of exogenous acyl-ghrelin increases alcohol craving^36^. This suggests that, in contrast to circuits that drive fear and depression, neural circuits for drug-seeking behavior remain responsive to ghrelin even when circulating ghrelin is elevated. The study of differential trafficking of ghrelin receptors in different brain regions is an important area for future research. In addition, the observation that one biological mechanism (elevated ghrelin) may contribute to multiple co-morbid mental disorders suggests that a single strategy (reducing circulating acyl-ghrelin) could have a multi-faceted therapeutic benefit.

Here, we use the phrase “chronic stress” to refer to a prolonged period of stress, but we acknowledge that precisely quantifying the duration of stress is difficult because stressors may continue to exert psychological effects in their physical absence. That is, mnemonic retrieval of stressful experiences, either consciously or subconsciously^37,38^, may continue to evoke a stress response which sustains the biological changes originally induced by the stressor itself. Because we cannot measure the spontaneous mnemonic retrieval of previous stressful experiences in rodents, we repeated the immobilization stressor to insure that the rats experienced stress for a minimum of 14 days. It is possible that either retrieval or replay of this experience after the cessation of stressor exposure adds to the cumulative duration of stress, but there are no methods to assess this. While a terror attack is often considered an acute event for humans, we selected the loss of a loved one and bodily injury as highly stressful and persistent consequences of terror attacks. A recent report found that adults rank “Death of a spouse/relative/friend” as the most stressful of 18 different life events; “Being seriously ill” was the fourth most stressful event^39^. As for rodents, it is extremely difficult to precisely assess the spontaneous mnemonic retrieval or subconscious replay of prior stressful events in humans. Thus, losing a loved one or experiencing bodily injury from a terror attack are more appropriately considered chronic stressors, albeit stressors of ill-defined duration. The extent to which such neural processes of stressor retrieval contribute to sustained acyl-ghrelin release remains to be determined.

We acknowledge children in the Control group do not represent an “unstressed” population. At a minimum, we can state that the children in the Traumatized group experienced a form of chronic stress that was not experienced by children in the Control group. We also acknowledge that the children in the Control group were likely to be aware of the terror attacks that impacted children in the Traumatized group, and terror attacks can increase self-reported stress levels in a subset of people who are geographically close to such attacks^40^. We made efforts to minimize or equate the influence of other stressor exposures across the Control and Traumatized groups. For example, we insured that all children had similar socioeconomic status because this can influence the type of stressors humans experience^41^. Additionally, we excluded all children who experienced multiple traumas from both groups. Perhaps reflecting these efforts, the large differences in sleep, emotional regulation, and social isolation that we observed between children in the Traumatized and Control groups (Table 2) suggest that any past stressors experienced by children in the Control group did not have the same persistent consequences as the consequences of the terror attacks experienced only by children in the Traumatized group.

Our results indicate that a protracted elevation of acyl-ghrelin mediates extended vulnerability to a PTSD-like phenotype in rats long after stressor exposure ceases (Fig. 3). Although a ghrelin receptor antagonist administered during 2 weeks of chronic stress is sufficient to prevent stress-enhanced fear when fear conditioning occurs 1 day after stressor exposure ceases^2^, we show here that adding a drug-free temporal gap between the cessation of stressor exposure and the start of fear conditioning permits the re-emergence of stress-enhanced fear. In contrast, if a ghrelin receptor antagonist was delivered throughout the entire period in which endogenous acyl-ghrelin was elevated by stressor exposure, stress-enhanced fear was not observed. It is of great interest to determine whether reducing ghrelinergic signaling throughout longer temporal windows after the cessation of stress prevents stress-enhanced fear at more remote time points. Ideally, such an experiment would use a specific GHSR1a antagonist with no known off-target activity, however the monetary cost of daily systemic drug delivery to rats and the labor-intensive nature of such an experiment are prohibitive. An alternative approach^42^ is to use gene transfer to overexpress butyrylcholinesterase, an enzyme that metabolizes acyl-ghrelin to its inactive form. In addition to enabling strong temporal control starting from any time point either during or following stress exposure, such an approach could also confirm that the periphery is the source of the acyl-ghrelin that regulates fear, as the gene transfer vectors do not enter the brain.

An additional important issue to address is whether there are gender differences in stress-related changes in acyl-ghrelin. Our rodent data have only examined male subjects, and the vast majority of the human subjects included here were male. Thus, it remains an open question of whether females show similar stress-dependent regulation of acyl-ghrelin.

Our study did not address the source of the acyl-ghrelin that is upregulated by chronic stress, nor the mechanism by which stress alters acyl-ghrelin. While multiple organs synthesize ghrelin, the stomach is thought to be the primary source of acyl-ghrelin in the bloodstream^43,44^. The mechanisms that regulate ghrelinergic tone from the stomach include vagal^45^ and sympathetic^46^ efferents; it is possible that chronic stress alters these inputs to the stomach to produce a persistent increase in acyl-ghrelin. Alternatively, it is possible that epigenetic changes in the ghrelin-producing cells of the stomach also play a role in the persistent changes in acyl-ghrelin that we report here.

Our study also did not address the downstream mechanisms by which elevated ghrelin leads to a PTSD phenotype. A previous study^3^ showed that acyl-ghrelin inhibits fear memory consolidation, and that chronic stress, via increased ghrelin signaling, leads to decreased central sensitivity to ghrelin. We speculate here that the continued elevation of acyl-ghrelin after the cessation of chronic stressor exposure produces a state of ghrelin resistance in the brain that persists throughout the period in which acyl-ghrelin remains elevated. Ghrelin resistance could either be driven by stress-induced increases in acyl-ghrelin crossing the blood–brain barrier^47^, or by stress-induced increases in des-acyl-ghrelin crossing the blood–brain barrier and becoming acyl-ghrelin in brain regions expressing ghrelin-O-acyltransferase^48^, the enzyme responsible for acylating ghrelin. Regardless, our results suggest that any such ghrelin resistance is not readily reversed, once induced, by pharmacological antagonism of the ghrelin receptor.

In conclusion, our data demonstrate the exceptional persistence of a stress-induced change in two mammals, and illuminate an important novel role for acyl-ghrelin in driving long-term vulnerability to heightened fear in a rodent model of stress-induced vulnerability to fear. Our findings add a new dimension to the claim that early-life adversity leads to vulnerability to disease by producing biological changes in the body that persist for years beyond the exposure to the stressor itself. Previous studies have largely focused on stress-induced changes in epigenetic markers^49^ and their downstream changes in gene expression^50^, particularly as they relate to genes involved in the classical “HPA” stress axis^51,52^. Our study is unusual because it identifies a persistently altered, stress-sensitive, and blood-based protein that is readily measured in venous blood samples. It remains of interest to determine whether increased acyl-ghrelin is a generalized response to chronic stress. This may be determined by examining whether other forms of intense stressor exposure, such as physical or emotional abuse, may also lead to elevated acyl-ghrelin. Collectively, our findings suggest a new biomarker that may facilitate the identification of individuals “at risk” for PTSD following exposure to a significant stressor, and suggest that pharmacological reduction of ghrelin signaling may provide longitudinal prophylaxis against PTSD in individuals exposed to prolonged stress.

## Electronic supplementary material

Supplemental Materials
