# Supplementary material for: Ghrelin is a persistent biomarker for chronic stress exposure in adolescent rats and humans
Source: Transl Psychiatry. 2018 Apr 11;8:74. doi: 10.1038/s41398-018-0135-5 (PMC5895712; doi:10.1038/s41398-018-0135-5)
Supplement: Supplementary file 1 — Supplemental Materials [file 41398_2018_135_MOESM1_ESM.doc]

**Supplementary Materials**

**Supplementary Materials and Methods**………………………………………..….pp. 2-7

**Figure S1**…………………………………………………………………………..….…p.8

**Figure S2**………………………………………………………………………….…..…p.9

**Figure S3**……………………………………………………………………….….……p.10

**Table S1**…………………………………………………………………………………p.11

**Questionnaire S1**………………………………………………………….….……pp.12-14

**Supplementary References**…………………………………………………………….p.15

**Supplementary Materials and Methods**

***Rodent subjects:***

Control animals were housed in separate cubicles from animals exposed to immobilization stress. Water and food was given *ad libitum*. Rats were pseudorandomly assigned to treatment groups such that the mean initial body weight was equivalent across the groups. All procedures involving rodents followed recommendations of the US National Institutes of Health (NIH) Guide for the Care and Use of Laboratory Animals.

***Human subjects:***

The researchers asked the Jirga to identify families with children who had either suffered the traumatic loss of a loved one or injury from a terrorist attack. Families with children who had not lost a loved one from such causes were recruited from the same neighborhoods as the children who had suffered loss or injury in a terror attack; researchers used social contacts to identify these families. Researchers then contacted the families and arranged for study visits at the home with interested parties. All evaluations and sample collection were performed at the home by the researchers. At the time of study participation, children were 14.31 ± 0.3 years of age, with a range of 8- 18 years. Children in the Traumatized and Control groups were recruited from the same socio-demographic background in a settled area of Peshawar.

***Chronic immobilization stress:***

For 14 consecutive days, stress-exposed rats were transferred from the animal facility to a behavioral testing room used only for stress at 11:00 AM, when immobilization stress began. They were returned to their home cages at 3:00 PM.

From 11:00 – 3:00 PM, unstressed control rats remained in their home cages without food and water, so that all animals experienced short-term food and water deprivation. This insured that any differences in acyl-ghrelin across groups could be attributed to difference in stress exposure, rather than differences in food availability.

***Rodent venous blood collection:***

Rats were not fasted prior to blood collection, but all samples were collected during the light cycle while rats remained in their home cages in the vivarium. Thus, most animals were sleeping immediately prior to sample collection. In addition, for rats, the vast majority of meals are consumed during the dark cycle^1^. Thus, while we anticipate that meal-associated changes in acyl-ghrelin were minimal across animals, we cannot rule this out as a source of variability in our measure.

Rats were restrained in a clean lab coat and the tail was warmed in water. A heparinized butterfly catheter (26 g) was used to withdraw roughly 500 µl of blood from the lateral tail vein into an eppendorf tube. The tube contained 10 µl of 0.1M EDTA and 4 µl of HALT cocktail (Thermo Scientific/Rockford, IL).

All samples were placed in tubes on ice until they were spun (2100 *g*, 4ºC, 10 min). The supernatant (plasma) was placed in new tubes. For analysis of acyl-ghrelin, 60 µl of plasma was placed in a tube containing 3 µl of 0.5M HCl. The remainder of plasma was placed in a second tube. All tubes were stored at -80ºC until analysis.

***Human venous blood collection:***

A solution of the protease inhibitor 4-(2-aminoethyl) benzenesulfonyl fluoride hydrochloride (AEBSF; 200 mg/ml) was prepared in distilled water and maintained on ice prior to sample collection. After consuming lunch at approximately 11:00 AM, subjects did not consume additional food until after the subsequent blood collection. Fasting venous blood samples were collected from the participants using disposable sterile syringes at approximately 5:00 PM. A small volume of blood (3 ml) was drawn by trained research staff. Within two minutes of blood collection, aliquots of blood (100 µl) were generated; AEBSF was added to each aliquot at a 1:100 dilution (i.e. 1 µl AEBSF per 100 µl of blood). Samples were gently inverted five times to mix completely, and then placed on ice. Serum was then collected from the clotted blood aliquots and stored at -80^o^C until analysis. We used serum samples for the analysis of acyl-ghrelin levels because we found that heparin or EDTA in pre-coated glass tubes used for human sample collection interferes with the ELISA detection of acyl-ghrelin.

***Human clinical evaluation:***

Sociodemographic details and past and current medical and psychiatric history were recorded for each child. The questions addressed changes in health status since the time of the terror attack that resulted in the death of a loved one or injury to the child (for children in the Traumatized group) and changes in the health status since the time of the natural death of a loved one or in recent years (if applicable, for children in the Control group). The questions also probed whether children were exposed to other traumas. Body Mass Index [BMI] was computed by dividing body mass (kg) by height squared (m^2^). This information was converted to an age-adjusted percentile using an online tool from the Division for Nutrition, Physical Activity, and Obesity at the Centers for Disease Control and Prevention (CDC; Atlanta, GA, USA) at https://nccd.cdc.gov/dnpabmi/Calculator.aspx?CalculatorType=Metric.

***Rodent fear conditioning***

Each rat was placed in a fear conditioning box (Med Associates; St. Albans, VT) scented with Pine-Sol (1%) for 13 min 50 sec total. The rats had 3 min to explore the novel environment before five tone (10 sec, 85 dB) habituation trials were presented [1 min intertrial interval (ITI)]. Five tone (10 sec, 85dB)-footshock (2 sec, 0.35mA; immediately following the tone) pairings [1 min ITI] were then administered. Long-term auditory fear memory was assessed 48 hr later. Auditory fear recall was administered by placing the rats in the fear conditioning boxes with an altered context. White Plexiglas inserts were used to obscure the back and side walls and cover the grid floor. Room and box lights were turned off, a red light provided illumination (15W), and acetic acid (1%) was used to scent the boxes. Two minutes were allowed for exploration before 4 tones (10 sec, 85 dB, 1 min ITI) were presented.

Infrared cameras (frame rate: 30Hz) and VideoFreeze software (Med Associates; St Albans, VT) were used to record behavior during all training and testing sessions. Freezing was measured by computing the number of observations below a threshold motion index and converting this number to a percentage of all observations for periods of interest. For a measurement to be scored as freezing, a minimum of 30 consecutive observations must have been below the threshold motion index; this means that freezing was only scored for periods lasting one second or greater. The threshold defined the value of the motion index below which only motor activity related to breathing was observed.

***Rodent study inclusion and exclusion criteria:***

For the study examining acyl-ghrelin in rats at a remote post-stressor exposure time point, three cohorts (biological replicates) of rats were run; this number of replicates was determined *a priori*. Each cohort contained rats pseudorandomly assigned to the Stressed and Control groups such that initial body weights were matched across the two groups. For the latter two cohorts, tail blood was collected one day following the last day of stressor exposure or handling, in addition to the remote (130 day post-stress) time point. If tail blood could not be collected with minimal stress (within two minutes of restraint, or two or fewer pokes with the butterfly catheter), the animal was returned to its home cage without sample collection; this criterion was determined *a priori*. At the 1 day post-stress time point, the experimenter was not able to collect tail blood from 6 rats with two or fewer needle pokes. For these six rats, blood was collected only at the 130 day time point. One rat died during immobilization stress; no data from this rat was included in any analysis.

For the study examining the role of ghrelin in stress-enhanced fear learning, three cohorts of rats were run; this number of replicates was determined *a priori*. Each cohort contained rats pseudorandomly assigned to each treatment group such that initial body weights were matched across the two groups. On the first day of fear conditioning, a camera malfunctioned in one conditioning chamber. This resulted in data not collected on this day for 6 rats. Rats were excluded from the study if high levels of conditional freezing (> 30%) were observed in the first two minutes after placement in the chamber for the auditory fear recall test; such freezing indicates inappropriate generalization of fear and precludes the accurate assessment of freezing specifically evoked by the auditory cue. Six rats met this *a priori* exclusion criterion, and were excluded from all analyses.

***Human study inclusion and exclusion criteria:***

An approximate minimum enrollment of n = 40 was selected for each group; this target group size was determined *a priori*. To be included in the Traumatized group, children must have lost a loved one (parent, relative or close friend) in a terror attack or been injured in a terror attack. To be included in the Control group, children must not have lost a loved one in a terror attack or been injured in a terror attack. In both groups, children who experienced, first hand, additional traumas (for example: multiple terror attacks, natural disasters such as earthquakes, disease epidemics, or parental abandonment) were excluded. Because the purpose of this study was to examine acyl-ghrelin levels following a severe stressor in otherwise healthy children, children with a previous diagnosis of a mental illness, including PTSD, or a developmental disorder were excluded from participation in the study. Study participants taking drugs which alter hormone levels, such as antidepressants, antipsychotic drugs or anti-anxiety drugs were also excluded from the study, as were children who had either previously suffered from significant illness (Chicken pox, Dengue Fever, etc.) or who currently showed signs of acute or chronic illness by physical examination. A total of 13 children were excluded from the study for these reasons; no data was included from these subjects in any analysis.

**
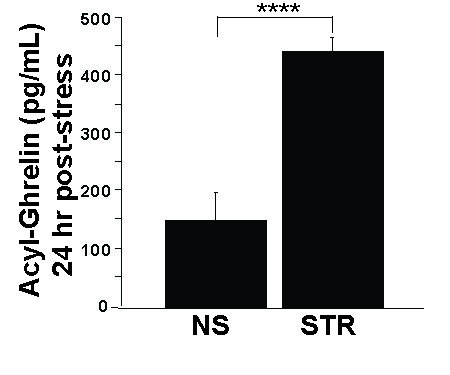
**

**Fig. S1**. Chronic stress in adolescent rats elevates acyl-ghrelin 24 hours post-stressor cessation. For a subset of the rats from Figure 1 (NS: n = 10; STR, group; n = 15), venous blood samples were collected 24 hr after the last session of immobilization stress or handling. Acyl-ghrelin was measured in the blood samples. All values are ±SEM. **** p < 0.0001 in a post-hoc comparison

**
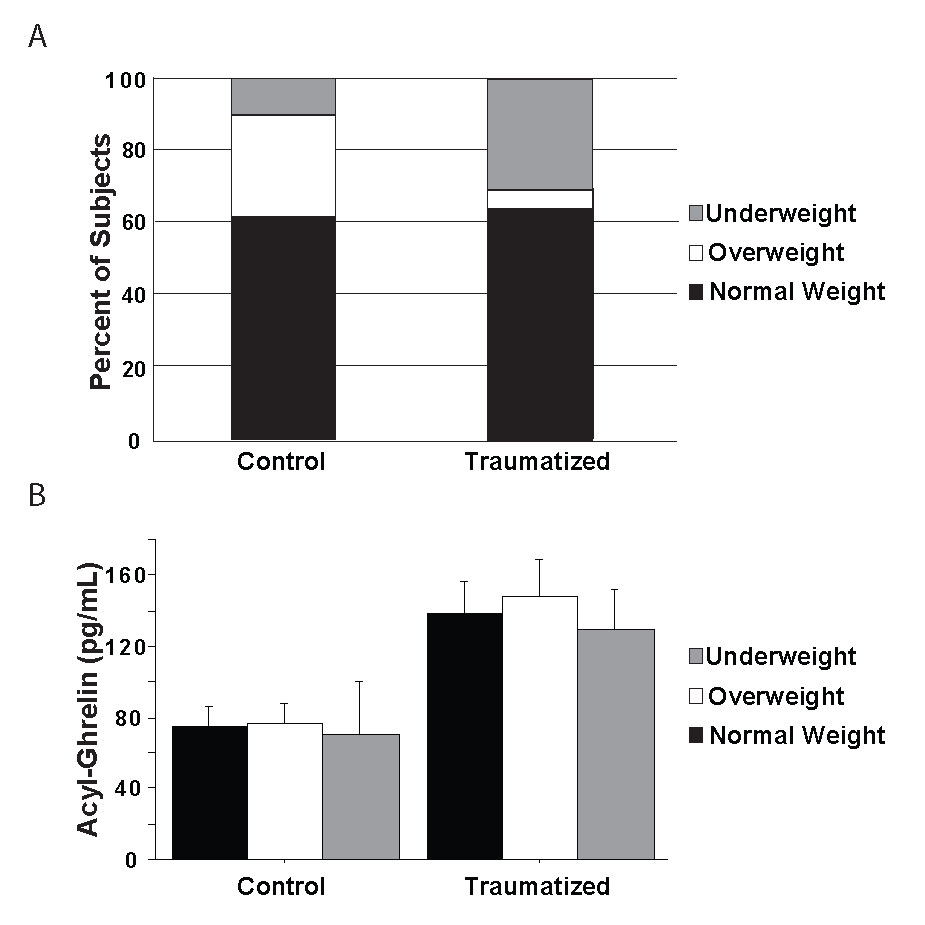
**

**Fig. S2**. BMI classification and acyl-ghrelin levels in adolescent human subjects. (**A**) There was a lower percentage of overweight and a greater percentage of underweight subjects in the Traumatized (n = 49) group as compared to the Control group (n = 39). However, the majority of subjects in both groups were classified within a normal weight range after adjustment for each subject’s age, gender, and height. (**B**) Mean acyl-ghrelin levels for each classification within the two groups are depicted. All values are ±SEM.

**
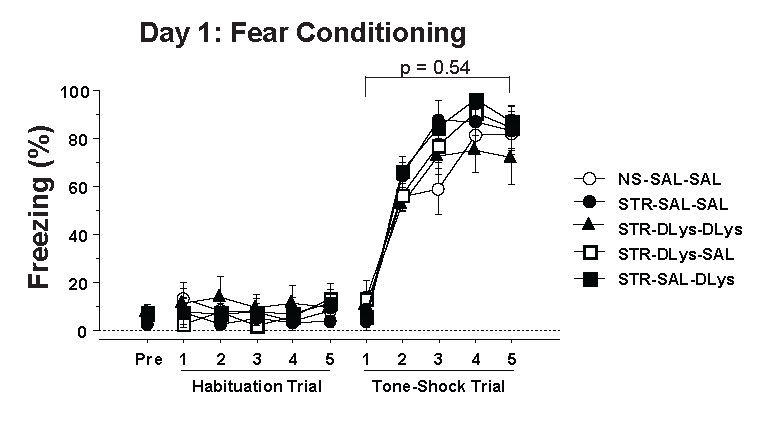
**

**Fig. S3.** Freezing during Pavlovian fear conditioning in rats. Freezing prior to auditory fear conditioning and during each 10 s tone presentation is depicted for the five treatment groups from Figure 3. All values are ±SEM. The p-value is from an ANOVA examining the Group X Trial effect for the tone-shock pairings administered during fear conditioning.

|  | Univariate | | Multivariate (R^2^= 0.17) | |
| --- | --- | --- | --- | --- |
|  | Beta | P-value | Beta | P-value |
| Group (Traumatized and Control) | 0.40 | 0.001 | 0.42 | 0.007 |
| Age at Study Initiation (yr) | 0.014 | 0.90 | 0.016 | 0.92 |
| BMI | -0.121 | 0.26 | 0.050 | 0.75 |
| Elapsed Time Since Loss of Closest Loved One | -0.075 | 0.60 | -0.041 | 0.77 |
| Socioeconomic status | 0.047 | 0.66 | 0.10 | 0.47 |

**Table S1.** Univariate and multivariate regression against acyl-ghrelin and other variables of interest.

**Questionnaire S1.**

**RESEARCH QUESTIONNAIRE PROFORMA FOR CHILD SURVIVORS OF TERROR ATTACKS (translated from Urdu to English)**

**Dated________________**

**Name of Subject ___________________________________ Age__________________ Sex_________________________ Weight_____________________________________**

**Occupation of father______________________________________________________**

**Address ________________________________________________________________**

**Date of Death of Loved One(s). ____________________________________________**

**Mode of Death__________________________________________________________**

**Any other relative injured?_________________________________________**

**PERSONAL HISTORY**

| Socioeconomic status of the family | School performance, before & after the incident | Health status before the incident | Any other current or past health condition | Taking any medication for PTSD |
| --- | --- | --- | --- | --- |
|  |  |  |  |  |

**Past history of other traumatic events or health conditions, if any. ________________________________________________________________________________________________________________________________________________**

**Treatment History (If YES, write the name of drug/dates in the respective column)**

| Antibiotics | NSAIDs | Antipsychotics | Antidepressants | Tranquilizers | Any other drug not listed here? |
| --- | --- | --- | --- | --- | --- |
|  |  |  |  |  |  |

| **Read carefully & tick mark the proper column** | | | |
| --- | --- | --- | --- |
| **S#** | **Question (for children in Traumatized group, ask relative to the date of the terror attack; for children in the Control group, ask relative to the last 5 years)** | **YES** | **NO** |
| 01 | Have your sleeping patterns changed considerably? |  |  |
| 02 | Has your weight changed considerably? |  |  |
| 03 | Have you experienced great or regular confusion? |  |  |
| 04 | Are there times when you been unable to stop crying? |  |  |
| 05 | Have you had difficulties concentrating? |  |  |
| 06 | Have others told you that you are not coping well? |  |  |
| 07 | Have you had suicidal thoughts? |  |  |
| 08 | Have you thought of harming yourself or others? |  |  |
| 09 | Have you been unable to make decisions? |  |  |
| 10 | Have you felt uncontrollable rage? |  |  |
| 11 | Have you experienced physical pain? |  |  |
| 12 | Have you been worried about your thoughts or behavior? |  |  |

| **Clinical Picture of Traumatized Children** | | | |
| --- | --- | --- | --- |
| **Physical** | | YES | NO |
| 01 | [Crying](http://www.webmd.com/a-to-z-guides/grief-crying-can-help-topic-overview) and sighing |  |  |
| 02 | [Headaches](http://www.webmd.com/migraines-headaches/default.htm) |  |  |
| 03 | Loss of appetite |  |  |
| 04 | Difficulty sleeping |  |  |
| 05 | Weakness |  |  |
| 06 | [Fatigue](http://www.webmd.com/a-to-z-guides/weakness-and-fatigue-topic-overview) |  |  |
| 07 | Feelings of heaviness |  |  |
| 08 | Aches, pains |  |  |
| **Emotional** | | YES | NO |
| 01 | Feelings of sadness and yearning |  |  |
| 02 | Worry, [anxiety](http://www.webmd.com/anxiety-panic/default.htm) |  |  |
| 03 | Frustration, anger |  |  |
| 04 | Guilt |  |  |
| **Social** | | YES | NO |
| 01 | Feeling detached from others |  |  |
| 02 | Isolating yourself from social contact |  |  |
| **Spiritual** | | YES | NO |
| 01 | Do you question the reason for your loss? |  |  |
| 02 | Do you think more about the purpose of life and the meaning of death? |  |  |

Supplementary References

1. Johnson RF, Johnson AK. Light/dark cycle modulates food to water intake ratios in rats. *Physiol Behav* 1990; **48**(5)**:** 707-711.
